# A Case of Pericarditis Caused by Human Herpesvirus 6 Infection Associated With Exanthema Subitem

**DOI:** 10.7759/cureus.78577

**Published:** 2025-02-05

**Authors:** Shuhei Fujita, Yoshikatsu Takeda, Yusuke Yachi, Takeshi Futatani, Yoshiki Kawamura

**Affiliations:** 1 Department of Pediatrics, Toyama Prefectural Central Hospital, Toyama, JPN; 2 Department of Pediatrics, Fujita Health University, Toyoake, JPN

**Keywords:** acute pericarditis, exanthema subitum, focal atrial tachycardia, human herpesvirus 6, infant, recurrent pericarditis

## Abstract

We encountered a case of a one-year-old girl who was diagnosed with focal atrial tachycardia (FAT) at two months old. The FAT was controlled with medical treatment. However, she later developed pallor and tachycardia, with a heart rate of 180 beats per minute (bpm). This occurred after an acute onset of high-grade fever for four days, followed by rapid fever reduction and a rash. She exhibited signs of chest discomfort by grabbing her clothes around her chest and complaining of chest pain. On the second day after the fever subsided, she suddenly became pale with tachycardia and was brought to our emergency department. A 12-lead electrocardiogram (ECG) revealed a sinus rhythm of approximately 120 bpm with frequent nonsustained FAT. Initial laboratory investigations showed normal results: creatinine kinase at 99 IU/L, troponin T at 0.010 ng/mL, and an elevated B-type natriuretic peptide level at 95.1 pg/mL. Echocardiography revealed a pericardial effusion of up to 6.6 mm despite normal cardiac function. We clinically diagnosed her with acute pericarditis and administered aspirin. The pericardial effusion resolved after two weeks but recurred two months later. Prednisolone was administered for recurrent pericarditis, and aspirin was replaced with colchicine. After one month, the pericardial effusion was resolved, and the prednisolone was discontinued. Subsequent echocardiography showed no pericardial effusion and no evidence of diastolic dysfunction. The quantitative polymerase chain reaction confirmed the presence of human herpesvirus 6 (HHV-6) in her serum at the onset of the disease. Additionally, serologic tests conducted in acute and chronic phases indicated viral antibody titers that were eight and 256 times higher, respectively. In conclusion, the HHV-6 virus can cause acute viral pericarditis in patients with exanthema subitum.

## Introduction

Exanthema subitum (roseola infantum, sixth disease) is caused by human herpesvirus 6 (HHV-6) infection, a benign febrile illness. Most children develop it in infancy [1]. Several fatal complications have been reported, including fulminant hepatitis, viral-associated hemophagocytic syndrome, Guillain-Barré syndrome, and encephalitis [1]. However, the condition is rarely associated with cardiac complications, such as myocarditis and pericarditis [2-4]. Acute pericarditis, along with acute myocarditis and infective endocarditis, is an inflammatory cardiac disease and the most common inflammatory heart condition [5-10]. It is caused by various factors, with 80%-85% of cases being infectious, most of which are viral [5-10]. Although pericarditis can be caused by various viral infections (enterovirus, coronavirus, influenza, herpesvirus, adenovirus, varicella, mumps, parvovirus B19, hepatitis B, hepatitis C, and HIV), HHV-6 is seldom linked to pericarditis, and there have been no reports of pericarditis occurring during exanthema subitum. This case report describes a one-year-old girl with acute pericarditis complicated by exanthema subitum due to HHV-6 infection.

## Case presentation

A one-year-old girl was brought to our emergency department at two months of age due to pallor and general unwellness. Her heart rate was measured at 225 bpm, and it was found that she had focal atrial tachycardia (FAT) originating from the right atrial appendage. Following hospitalization, her heart returned to a normal rhythm with the administration of atenolol (1.5 mg/kg/day) and ivabradine (0.34 mg/kg/day). While being treated for FAT, there were no signs of pericardial effusions or worsening of cardiac function in her echocardiography scans. To detect FAT early at home, we trained her mother to auscultate this patient’s heartbeat during hospitalization. After being discharged, her mother checked the girl’s heart rate 2-3 times daily for signs of tachycardia but observed no tachycardia accompanied by pallor.

When she was one year old, she visited a local pediatrician because she had a high fever and cough. The fever lasted four days and then suddenly disappeared, leaving a rash mainly on her trunk. On the fourth day of the fever, she sometimes seemed to be in pain in her chest. The second day after the fever went away, she was tachycardic (approximately 180 bpm) and looked pale, so we took her to the emergency department. When we arrived at the hospital, she was asymptomatic but in pain. Her body temperature was 37.1°C, her heart rate was 124 bpm, and her blood oxygen level was 100%. She was 68.0 cm (−2.2 SD) tall and weighed 7485 g (−1.2 SD). She appears smaller than average for a one-year-old but has no other abnormalities and develops normally. We didn't hear any unusual sounds when we listened to her heart. Her lab results showed a white blood cell count of 5,800 cells /mm^3^, creatine kinase of 99 (MB 3) IU/L, C-reactive protein of 0.17 mg/dl, brain natriuretic peptide of 95.1 pg/ml, and troponin T of 0.010 ng/mL (Table 1).

**Table 1:** Laboratory investigations ANA: antinuclear antibody; Alb: albumin; ALT: alanine transaminase; AST: aspartate transaminase; BE: base excess; BNP: brain natriuretic peptide; BUN: blood urea nitrogen; Ca: calcium; Cl: chlorine; CK: creatine phosphokinase; Cre: creatinine; CRP: C-reactive protein; γ-GT: γ-glutamyl transpeptidase; Glu: blood glucose; F-T3: free triiodothyronine; F-T4: free thyroxin; HHV: human herpesvirus; Ht: hematocrit; Hb: hemoglobin; Ig: immunoglobulin; K: potassium; Lac: lactate; LDH: lactate dehydrogenase; Lym: lymphocytes; Mg: magnesium; Mono: monocytes; Na: sodium; Neut: neutrophils; Plt: platelet; PVB19: parvovirus B19; RBC: red blood cells; RF: rheumatic factor; qPCR: quantitative polymerase chain reaction; RT-PCR: real-time polymerase chain reaction; SARS-CoV-2: severe acute respiratory syndrome coronavirus 2; T-Bil: total bilirubin; TP: total protein; TSH: thyroid-stimulating hormone; WBC: white blood cells

Lab test	Result	Unit	Lab test	Result	Unit	Lab test	Result	Unit	Lab test	Result	
WBC	5800	/μL	TP	6.5	mg/dL	pH	7.335		Interferon-gamma release assay	(-)	
Neu	26	％	Alb	4.4	mg/dL	PCO2	36.7	mmHg	ANA	40	
Seg	26	%	AST	53	IU/L	HCO3	19.1	mg/dL	RF	(-)	
Eos	1	%	ALT	29	IU/L	BE	-5.7	mmol/L	PVB19 IgM	(-)	
Lym	65	％	LDH	427	IU/L	Glu	95	mg/dL	Throat (RT-PCR)		
Mon	8	％	CK	99	IU/L	Lac	2.3	mmol/L	Parechovirus	(-)	
RBC	485 x 104	/μL	CK-MB	3	ng/mL	BNP	95.1	Pg/mL	PVB19	(-)	
Hb	12.6	g/dL	γ-GT	9	U/L	Troponin T	0.01	ng/mL	Enterovirus	(-)	
Ht	38.2	％	T-Bil	0.5	mg/dL	IgG	606	mg/dL	Rhinovirus	(-)	
Plt	14.1 x 104	/μL	BUN	12	mg/dL	IgA	34	mg/dL	HHV-6	(+)	
			Cre	0.23	mg/dL	IgM	103	mg/dL	HHV-7	(-)	
			Na	138	mEq/L	TSH	2.67	mIU/L			
			K	4.9	mEq/L	F-T3	3.5	pg/dL	Serum (HHV-6 qPCR, IgG titer):	Result at 7 days	Result at 1 month
			Cl	108	mEq/L	F-T4	1.2	ng/dL	qPCR (copies/ml)	53600	0
			CRP	0.17	mg/dL				IgG titer	8	256＜

**Figure 1:**
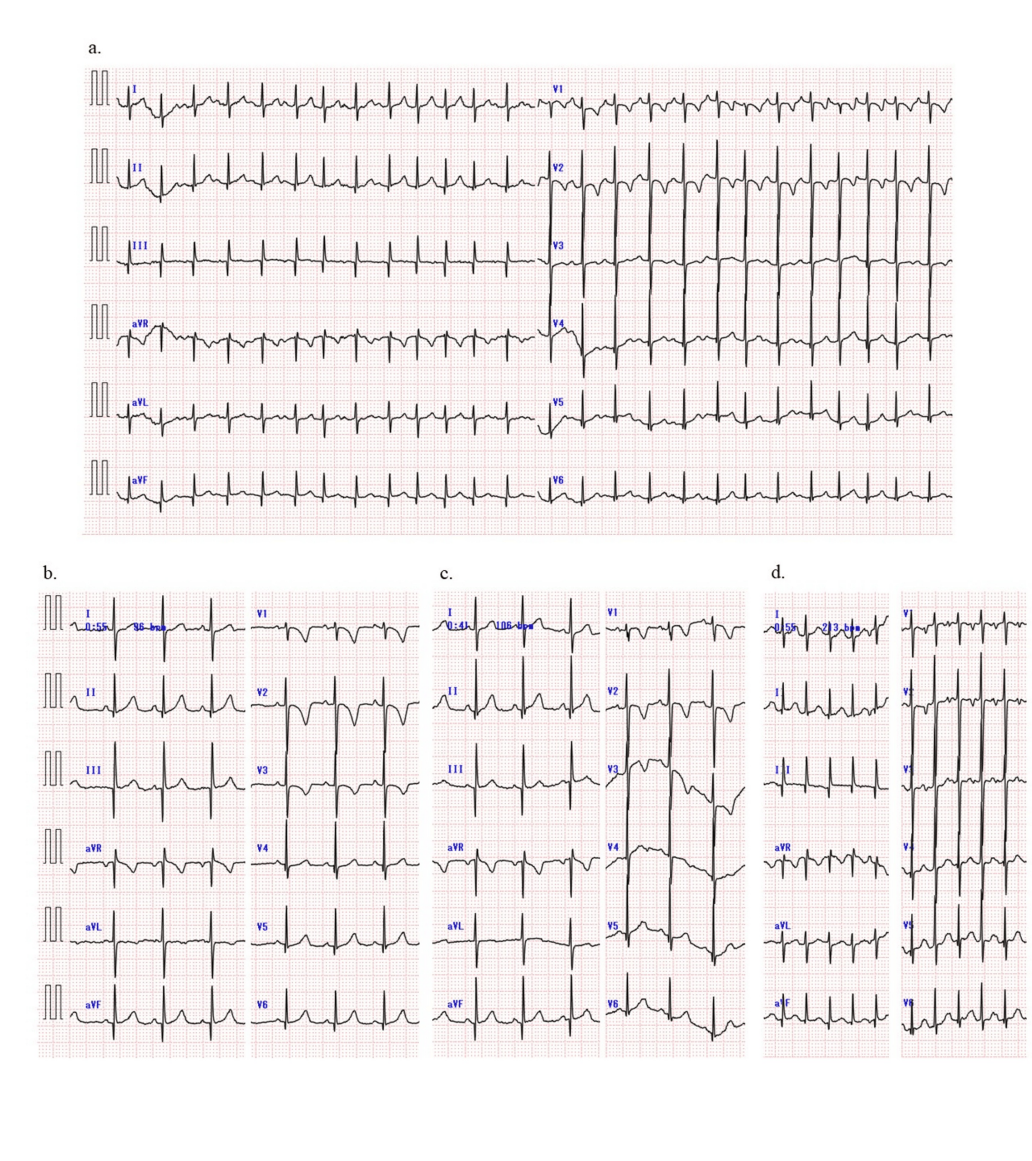
Electrocardiogram findings ECG: electrocardiogram; FAT: focal atrial tachycardia; PR: pulse rate; aVF: arteriovenous fistula (a) The 12-lead electrocardiogram depicts the presence of atrial extrasystoles and nonsustained atrial tachycardia in sinus rhythm. No signs of low potential, low PR, or ST-segment elevation exist deemed specific for pericarditis, but T wave flattening is observed in leads III and aVF. (b) Normal ECG during an outpatient visit two months prior. (c) ECG changes normalized after one month. (d) Nonsustained FAT at the recurrence of pericarditis

The chest X-ray showed no sign of lung congestion and consolidations, and the cardiothoracic ratio was 52%, compared to 47% at the regular outpatient visit two months earlier. The 12-lead electrocardiogram (ECG) indicated sinus rhythm, nonsustained FAT, and a flat T wave on lead III and aVF, unlike the results from the outpatient visit two months prior (Figures 1a, 1b).

**Figure 2:**
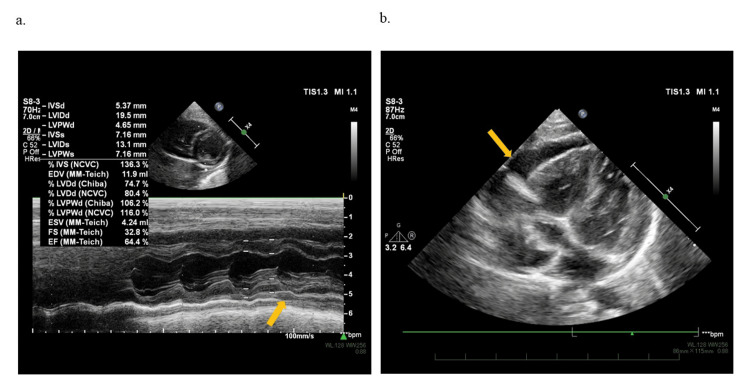
Two-dimensional echocardiography findings (a) Normal left ventricular ejection fraction throughout the cardiac cycle, with evidence of pericardial effusion. (b) Pericardial effusion measuring up to 6.6 mm visible in front of the right atrium and right ventricle

An ECG revealed normal cardiac function with a left ventricular ejection fraction of 64%, but it also showed a circumferential echo-free space (6.6 mm), particularly anterior to the right atrium and right ventricle (Figures 2a, 2b).

Based on the appearance of a rash after four days of fever, chest pain during fever, FAT with flat T wave that was not peculiar to the pericarditis on ECG, and pericardial effusion with normal cardiac function on echocardiographic results, she was clinically diagnosed with acute pericarditis complicated by exanthema subitum. Since there were no symptoms of pericardial tamponade, no impaired cardiac function, and the pericardial effusion was too small for drainage, the medical team initiated treatment with aspirin (30 mg/kg/day). Although nonsteroidal anti-inflammatory drugs are commonly used to treat acute pericarditis in pediatric patients, we began with aspirin, which has been used often in post-pericardiotomy syndrome, Kawasaki disease, and other conditions. The following day, she was able to take aspirin. She had no symptoms, and her ECG revealed normal cardiac function and a decreased pericardial effusion (4.4 mm). After 14 days of aspirin administration, a follow-up ECG showed a decrease in pericardial effusion (3.2 mm), and by day 28, the effusion had disappeared, with a normalized 12-lead ECG (Figure 1c).

**Figure 3:**
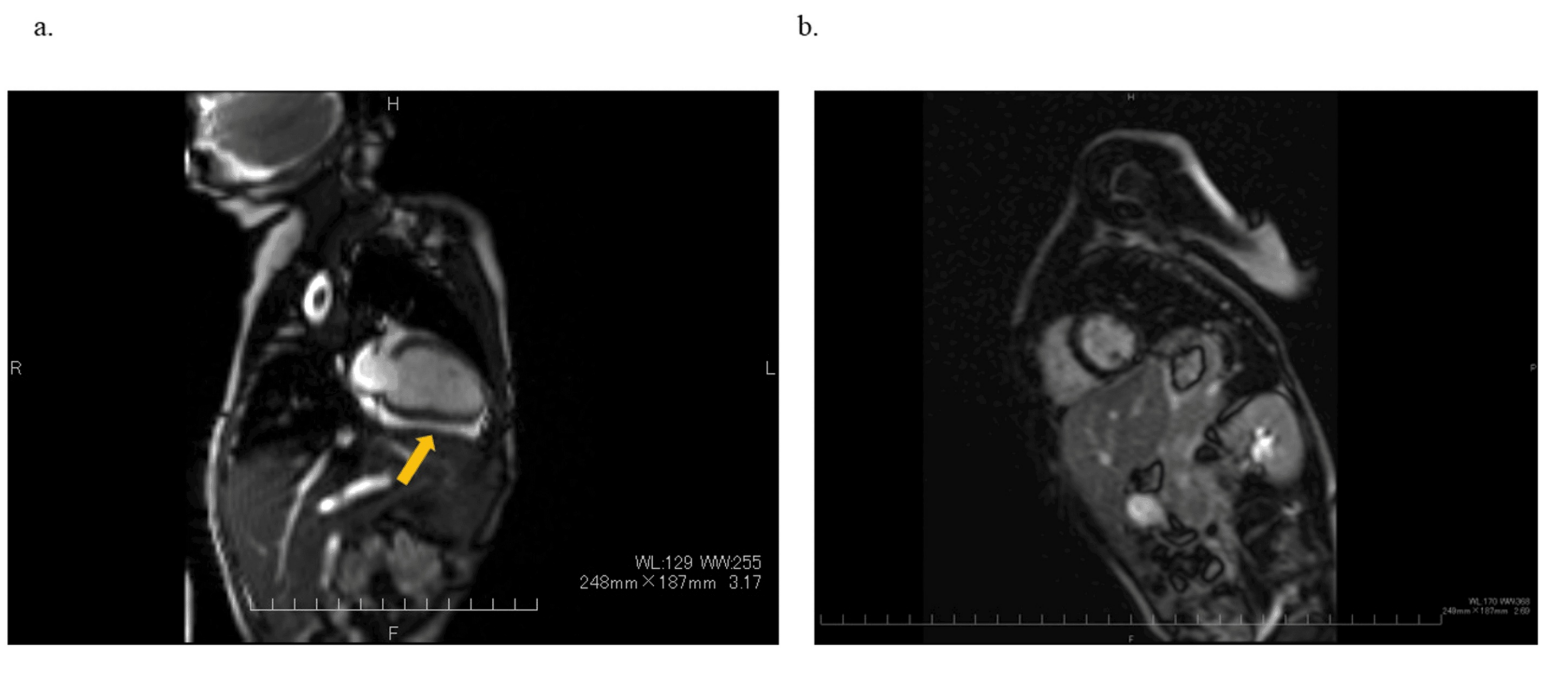
Cardiac MRI MR: magnetic resonance; LGE: late gadolinium enhancement; SSPF: steady-state free precession A cardiac MRI obtained after the recurrence of pericardial effusion demonstrates the presence of pericardial effusion without any evidence of LGE in the myocardium or pericardium. (a) SSPF image depicting pericardial effusion and the absence of myocardial edema. (b) The LGE image displays no enhancement in the myocardium or pericardium

She did not show any symptoms after taking aspirin for two months. However, on the day of her outpatient visit, she complained of chest pain that worsened when lying down but improved when sitting up. Irregular heart sounds were heard, and a 12-lead ECG revealed nonsustained FAT (Figure 1d). Pericardial effusion (5.0 mm) recurred during an echocardiography. Since it had been two months since the onset of the disease and the recurrence occurred after the pericardial effusion had disappeared, she was started on prednisolone (1 mg/kg/day) for recurrent pericarditis as an anti-inflammatory therapy, and aspirin was replaced with colchicine (0.01 mg/kg/day). Cardiac magnetic resonance imaging was performed and showed pericardial effusion but no late gadolinium enhancement (LGE) of the myocardium or pericardium (Figures 3a, 3b).

After receiving prednisolone and colchicine, the patient’s pericardial effusion decreased (2.7 mm; six days after recurrence), and her chest pain improved quickly. The prednisolone dosage was gradually reduced and stopped after about one month (1 mg/kg/day for five days; 0.5 mg/kg/day for five days; 0.2 mg/kg/day for 14 days; 0.1 mg/kg/day for 14 days). Subsequently, colchicine was continued for six months due to recurrent pericarditis and then stopped. After stopping the treatment, there was no recurrence of pericardial effusion, and the patient is currently being monitored as an outpatient. After discontinuing medical therapy, she was treated with FAT once every three months and monitored as an outpatient for 10 months, with no recurrences. Follow-up echocardiography showed no signs of diastolic heart failure. Pulse-wave Doppler ECG showed a normal flow pattern in the mitral and tricuspid valves. Tissue Doppler imaging also indicated a normal mitral E/e’ ratio.

We investigated the causes of pericarditis in pediatric patients. Infection is often the cause of pericarditis, with tuberculosis being the most common. It is now more common after viral infections, and polymerase chain reaction (PCR) tests have been performed on the main viruses. In addition, tests were performed to rule out pericarditis caused by rheumatic diseases. In other laboratory investigations, tuberculosis was ruled out through interferon-gamma release assays. Antinucleotide antibodies and rheumatic factor tested negative. The pharyngeal swab virus real-time PCR detected HHV-6 at the onset, and the serum quantitative PCR showed 53,600 copies/ml at the onset and 0 copies/ml in the chronic phase. Serological examination revealed an eightfold increase in HHV-6 IgG titer during the acute phase and a 256-fold or higher increase in HHV-6 IgG titer during the chronic phase (Table 1).

## Discussion

We treated an infant with acute pericarditis complicated by exanthema subitum, which is associated with HHV-6 infection. To the best of our knowledge, this is the first documented case of an infant developing acute pericarditis during exanthema subitum as a result of HHV-6 infection. The infant’s condition improved after receiving medical treatment that avoided invasive procedures such as drainage or pericardiectomy. However, after the pericardial effusion resolved with aspirin alone, a recurrence was observed. The recurrence could have been avoided if colchicine had been used concurrently, as recommended in adult treatment protocols [5-7]. During the acute phase, a 12-lead ECG showed T wave flattening and recurrent nonsustained FAT. Echocardiography revealed pericardial effusion. Clinically, the patient showed typical symptoms of exanthema subitum. HHV-6 was detected in pharyngeal swabs and serum during the acute phase. Serologically, the patient tested negative for IgG antibody in the acute phase and positive for antibody titer in the chronic phase. Although exanthem subitum is a benign infantile febrile illness, it is critical to rule out cardiac complications in atypical presentations such as chest pain, tachycardia, prolonged feeling of unwellness, and prolonged low-grade fever.

The cause of acute pericarditis due to HHV-6 infection is not well understood. However, it has been reported that the HHV-6 virus has an affinity for the myocardium [11]. Previous cases of acute myocarditis, acute pericarditis, and constrictive pericarditis due to HHV-6 in pediatric patients are presented in Table 2 [3,4,12-17].

**Table 2:** Acute myo- and pericarditis and constrictive pericarditis due to HHV-6 in pediatric patients HCT:  hematopoietic cell transplantation; HHV-6:  human herpes virus 6; HUS: hemolytic uremic syndrome; PSL: prednisolone; PVB19: parvovirus B19; STEC: Shiga-like toxic-producing *Escherichia coli*; Ref: reference number

Author/year/Ref	Age/Sex	Symptom	Immunosuppression	Virus	Diagnosis	Therapy	Outcome
Yoshikawa, 2001 [3]	5 m/female	Lethargy, poor feeding	Healthy	HHV-6 (B)	Exanthema subitum, acute myocarditis	Medical treatment (diazepam, disopyramide)	Death
Rohayem, 2001 [4]	11 y/male	Acute distress	Healthy	HHV-6 (B), PVB19	Erythema infectiosum, acute myocarditis	Intubation, catecholamines	Death
Stefanski, 2016 [12]	13 y/male, 2 y/female	Fever, general erythroderma, decreased urine output, change in mental state, hypotension	HCT for leukemia PSL for Evans syndrome	HHV-6 (A) HHV-6 (A)	Acute myocarditis, acute myocarditis	Ganciclovir, none	Death, death
Papadopoulou-Legbelou, 2016 [17]	41 d/female	Fatigue during feeding	Healthy (neonate)	HHV-6	Acute myocarditis, DCM	Inotropies, diuretics, anti-arrhythmic drug	Live
Backhoff, 2013 [15]	9 y/female	Abdominal girth, exercise intolerance	Healthy	HHV-6, PVB19	Constrictive pericarditis	Open pericardectomy	Live
Mounier, 2021 [17]	27 m/female	Diarrhea, rush, convulsion	Healthy	STEC, HHV-6B	HUS, encephalitis, acute pericarditis	colchicine Pericardial drainage	Live
This case, 2023	1 y/female	Chest pain, tachycardia	Healthy	HHV-6	Exanthema subitum, acute/recurrent pericarditis	Aspirin, prednisolone, colchicine	Live

Acute myocarditis caused by HHV-6 appeared to have a worse prognosis than acute pericarditis, but there were no significant differences in prognosis based on gender, age, underlying conditions, or treatments. Acute pericarditis was more prevalent during HHV-6B-induced exanthema subitum than acute pericarditis caused by HHV-6A reactivation. There are two presumed mechanisms: one is a complication after acute myocarditis, and the other is direct infiltration of the pericardium [3,4,12-17]. Acute myocarditis developing during HHV-6 infection has often been severe and fatal in previous reports [3,4,12-14]. However, the clinical course of this patient was thought to be different. In a case of direct infection of the pericardium, HHV-6 was detected in drained pericardial fluid but not in serum in an adult patient with acute pericarditis who underwent cord blood transplantation, suggesting that acute pericarditis was caused by local reactivation of HHV-6 in an immunosuppressed state [13]. However, in this patient, it is difficult to know the mechanism of acute pericarditis because myocardial biopsy by cardiac catheterization was not performed, and pericardial effusion was not punctured and drained. Pericardial drainage and myocardial biopsy are important tests for determining the relationship between HHV-6 infection and pericarditis or myocarditis. However, we did not drain a pericardial effusion that had not yet progressed to cardiac tamponade or perform a myocardial biopsy on an infant with no evidence of myocarditis because we thought these procedures were overly invasive. According to the clinical course and cardiac MR, we considered direct infection for the pericardium to be the likely pathogenesis of this patient.

This patient had a history of FAT and recurrent nonsustained FAT at the onset and recurrent pericarditis so acute pericarditis could be diagnosed by 12-lead ECG and echocardiography. The FAT at the onset or recurrence of pericarditis also pointed to the right atrial appendage. We hypothesized that the pericardial inflammation spread to the atrial myocardium, resulting in FAT via an automaticity mechanism. Acute pericarditis is a benign and self-limited disease [5-10]. Backhoff et al. reported on a nine-year-old girl with severe constrictive pericarditis in which the genomes of parvovirus B19 and HHV-6 were detected in a pericardial section. In this report, a history of erythema infectiosum and exanthem subitum was 1-2 years of age [14]. Although there have been no reports of acute pericarditis in exanthema subitum, some patients are asymptomatic and may resolve spontaneously. For accurate diagnosis, it is essential to be aware that acute pericarditis can be associated with exanthema subitum. Symptoms such as chest pain should not be overlooked. Correct diagnosis of acute pericarditis with exanthema subitum and follow-up are crucial because some patients may develop cardiac tamponade, recurrent pericarditis, or constrictive pericarditis.

## Conclusions

In conclusion, HHV-6 can cause viral acute and recurrent pericarditis in patients with exanthem subitum. It is important to diagnose and provide medical treatment to prevent complications accurately. To make an accurate diagnosis, it is critical to recognize that acute pericarditis can coexist with exanthema subitum. Symptoms such as chest pain should not be ignored. Exanthem subitum is a benign infantile febrile illness, and while acute pericarditis is extremely uncommon, an ECG and echocardiography should be carried out to rule out cardiac complications in atypical presentations such as chest pain, tachycardia, prolonged not feeling well, and prolonged low-grade fever.
